# Improving the characterization of *ex vivo* human brain optical properties using high numerical aperture optical coherence tomography by spatially constraining the confocal parameters

**DOI:** 10.1117/1.NPh.7.4.045005

**Published:** 2020-10-21

**Authors:** Jiarui Yang, Ichun Anderson Chen, Shuaibin Chang, Jianbo Tang, Blaire Lee, Kıvılcım Kılıç, Smrithi Sunil, Hui Wang, Divya Varadarajan, Caroline Magnain, Shih-Chi Chen, Irene Costantini, Francesco Pavone, Bruce Fischl, David A. Boas

**Affiliations:** a Boston University, Department of Biomedical Engineering, Boston, United States; b Boston University, Department of Electrical and Computer Engineering, Boston, United States; c Massachusetts General Hospital, A.A. Martinos Center for Biomedical Imaging, Department of Radiology, Boston, United States; d The Chinese University of Hong Kong, Department of Mechanical Engineering, Hong Kong Special Administrative Region, China; e University of Florence, European Laboratory for Non-Linear Spectroscopy, Sesto Fiorentino, Florence, Italy; f National Research Council, National Institute of Optics, Italy; g Health Science and Technology/Computer Science & Artificial Intelligence Laboratory, Massachusetts Institute of Technology, Cambridge, Massachusetts, United States

**Keywords:** optical coherence tomography, human brain tissue, index matching

## Abstract

**Significance:** The optical properties of biological samples provide information about the structural characteristics of the tissue and any changes arising from pathological conditions. Optical coherence tomography (OCT) has proven to be capable of extracting tissue’s optical properties using a model that combines the exponential decay due to tissue scattering and the axial point spread function that arises from the confocal nature of the detection system, particularly for higher numerical aperture (NA) measurements. A weakness in estimating the optical properties is the inter-parameter cross-talk between tissue scattering and the confocal parameters defined by the Rayleigh range and the focus depth.

**Aim:** In this study, we develop a systematic method to improve the characterization of optical properties with high-NA OCT.

**Approach:** We developed a method that spatially parameterizes the confocal parameters in a previously established model for estimating the optical properties from the depth profiles of high-NA OCT.

**Results:** The proposed parametrization model was first evaluated on a set of intralipid phantoms and then validated using a low-NA objective in which cross-talk from the confocal parameters is negligible. We then utilize our spatially parameterized model to characterize optical property changes introduced by a tissue index matching process using a simple immersion agent, 2,2’-thiodiethonal.

**Conclusions:** Our approach improves the confidence of parameter estimation by reducing the degrees of freedom in the non-linear fitting model.

## 1 Introduction

Optical coherence tomography (OCT) is a volumetric imaging technique that has been widely used to image the microstructure of biological samples.^1^ The OCT signal originates from back-scattered photons and provides image contrast that reflects intrinsic differences in tissue properties within the sample. The depth profile of the OCT signal is determined by five factors: the refraction index of the sample, the optical attenuation coefficient, the back-scattering coefficient, the numerical aperture (NA) of focusing optics, and the depth of the focus in the sample.^2^ The attenuation coefficient, i.e., the sum of the tissue scattering and absorption coefficients, is a spatially varying intrinsic property of the sample and is independent of the optics and the incident power of the system. For low-NA OCT systems, where the lateral resolution is typically larger than 10  μm and the confocal parameter (i.e., two times the Rayleigh range) is comparable to or larger than the imaging depth, a single-scattering exponential model is appropriate to describe OCT signal attenuation versus depth.^3^^,^^4^ Previous studies have shown that extracting the attenuation coefficient from low-NA OCT measurements based on Beer’s law is useful in quantifying the optical properties of the sample for applications, including detecting cancerous tissue in skin, bladder, and brain,^5^^–^^7^ monitoring blood glucose concentration,^8^ characterizing atherosclerosis plaques^3^^,^^9^ and correlating collagen content with histological staining.^10^ However, for high-NA OCT systems where the lateral resolution reaches a few microns or less, the shape of the axial point spread function (PSF) plays an important role in modulating the OCT signal depth profile, and thus the effect of the confocal parameter and focus depth cannot be neglected when estimating the optical properties of the sample.^4^^,^^11^ We have previously shown that OCT with a 3.5-μm lateral resolution is able to differentiate laminar structures in the neocortex of human brain samples using the average intensity projection (AIP) over the effective Rayleigh range.^2^^,^^12^ Moreover, we showed that estimating the intrinsic optical properties of the sample could provide additional information to differentiate brain structures, albeit with high inter-parameter correlations that resulted in large variance.^2^

Extensive mapping of brain structures and neuronal connections in postmortem human brain requires high-throughput microscopic imaging over large volumes. One major limitation is penetration depth due to tissue light scattering.^13^ Different approaches have been developed to overcome this challenge, among which optical clearing agents are suitable for various applications and imaging modalities.^14^ Water-based optical clearing reagents, such as Scale,^15^ See Deep Brain,^16^ ClearT,^17^ and Clear Unobstructed Brain/Body Imaging Cocktail and Computational analysis,^18^ are designed to increase the penetration depth in fixed brain samples while allowing for improved preservation of fluorescence.^19^^,^^20^ Most of them, however, present other nonnegligible limitations, such as long incubation time, severe structural alteration, and incompatibility with immunostaining. A glycol derivative, 2,2′-thiodiethanol (TDE), was previously reported to be a versatile, rapid, and inexpensive water-soluble clearing agent that overcomes these limitations.^21^^,^^22^ However, the impact of TDE optical clearing via index matching on the optical property contrast that permits imaging of brain structures by OCT has not yet been well studied.

In this work, we develop a systematic approach to spatially parametrize the confocal parameters across a three-dimensional (3-D) OCT image to constrain and reduce the degrees of freedom in the nonlinear coefficient fitting problem, resulting in improved confidence in the estimated optical properties of the sample. We first evaluate our spatially parametrized model on Intralipid phantoms with varied scattering levels. We compare the estimated coefficients using a high-NA objective with a low-NA objective to validate our parametrized model. We then quantify the improvements afforded by our model compared with the previously established model that does not employ spatial parameterization.^2^ Finally, we utilize our fitting procedure to characterize the optical property changes of a human brain motor cortex sample under index matching in TDE. We quantify the reduction in the scattering coefficients of both gray matter (GM) and white matter (WM) with index matching, and show differential responses of GM versus WM with the concentration of TDE.

## 2 Method

### 2.1 Theoretical Model

Based on Schmitt et al.^4^ and Izatt et al.,^11^ the derived single scattering model of the OCT signal can be written as

$$A(z)=\frac{\eta e\tau_{i}P_{0}}{2\;hv}\sqrt{R(z),}$$ (1)

where $P_{0}$ is the incident power, η is the quantum efficiency of the camera sensor, e is the electron charge, $\tau_{i}$ is the integration time of the line-scan camera, and hv is the photon energy. R(z) is the reflectance from the sample at the depth of z, and z=0 is the tissue surface. The reflectance is determined by two factors: the back-scattering coefficient ($\mu_{b}$) and the total attenuation coefficient ($\mu_{t}$), which is the sum of the scattering coefficient ($\mu_{s}$) and the absorption coefficient ($\mu_{a}$). In the near-infrared range, light attenuation within the tissue is dominated by scattering and light absorption is negligible. Therefore, we use $\mu_{s}$ to represent the total attenuation coefficient in this paper. Further, due to the shallow depth range of the OCT measurement, we can generally neglect the effects of multiply scattered light.^23^ Both $\mu_{b}$ and $\mu_{s}$ are assumed constant over depth in one OCT A-line (an assumption we hope to remove in the future). The OCT reflectance signal versus depth can thus be expressed as

$$R(z)=\mu_{b}\cdot \exp(-2\mu_{s}z)\cdot h(z),$$ (2)

where h(z) is the axial PSF, which is dependent on the refractive index of the medium (n), the focus depth ($z_{f}$), and the Rayleigh range ($z_{R}$). In a single-mode fiber-based OCT system, the light beam in the sample arm approximately follows a Gaussian distribution.^24^ Since we measure our tissue samples under liquid immersion, a simplified PSF model that neglects the effect of the refractive index difference at the tissue-air interface is given as

$$h(z)=\frac{1}{1+{\left(\frac{z-z_{f}}{n\cdot z_{R}}\right)}^{2}}=\frac{1}{1+{\left(\frac{z-z_{f}}{z_{r}}\right)}^{2}},$$ (3)

where $z_{r}=n\cdot z_{R}$ is the effective Rayleigh range. The Rayleigh range is calculated given the optics of the system as

$$z_{R}=\frac{\pi \omega_{0}^{2}}{\lambda_{0}}\approx \frac{\omega_{0}}{NA},$$ (4)

where $\omega_{0}$ is the beam waist at the focus, $\lambda_{0}$ is the center wavelength of the laser, and NA is the numerical aperture of the optics. However, in a highly scattering medium measured with a high-NA objective, the effective Rayleigh range is observed to deviate from the theoretical value as a function of $z_{f}$ and $\mu_{s}$^25^

$$z_{r}^{\prime }=z_{r}\cdot \sqrt{1+\frac{\mu_{s}z_{f}}{4}{\left(\frac{NA}{n}\right)}^{2}.}$$ (5)

This occurs because the oblique rays with longer path lengths are attenuated more than the on-axis rays, which effectively reduces the NA of the focusing optics. In our set up with NA=0.28, this effect is negligible. In our fitting model, $z_{r}$ is assumed to be constant over the entire image.

The focus depth, on the other hand, is not constant over the image because of two confounding effects. First, we must correct for the field curvature effect that arises from the different path lengths that light travels to the image focus for different positions in the image. We calibrated this field curvature correction for our system by imaging a mirror placed at the image focus.^26^ The second confounding effect is the likelihood that the surface of the brain sample being imaged is not parallel with the image plane. To correct for this effect, we parameterize $z_{f}$ within the imaging field of view (FOV) as

$$z_{f}(x,y)=A+Bx+Cy,$$ (6)

where x and y correspond to positions in the image as this accounts for the possibility that the image plane is not parallel to the surface of the sample.

Finally, the back-scattering coefficient $\mu_{b}$ can be quantified with known η and $P_{0}$. However, this would require knowledge of optical losses of all optical components in the OCT system. A relative back-scattering coefficient was previously introduced as

$$\mu_{b}^{\prime }={\left(\frac{\eta e\tau_{i}P_{0}}{2hv}\right)}^{2}\mu_{b}.$$ (7)

This relative back-scattering coefficient is system dependent and could be used to estimate the relative spatial variations in $\mu_{b}$.^2^

### 2.2 Spectral-Domain OCT System

We used a commercial spectral-domain OCT system (Telesto TEL320C1, Thorlabs, New Jersey) to measure the optical properties of human brain tissue. The light source is a broadband superluminescent diode with center wavelength of 1300 nm and a full width half maximum bandwidth of 150 nm, yielding an axial resolution of 4.2  μm in tissue. The spectrometer has a 2048-pixel InGaAs line scan camera operating at an A-line rate of 76 kHz. The total imaging depth is 2.6 mm in tissue. A 10× air objective (Mitutoyo) was used in the sample arm, which yields a lateral resolution of 3.5  μm with a theoretical Rayleigh range of 40  μm in a nonscattering medium. The maximum sensitivity of the system is 109 dB.

In spectral-domain OCT, the sensitivity drops with imaging depth due to the spectrometer resolution and discrete spectral sampling by the line-scan camera. The sensitivity roll-off function is given as^27^

$$H(z)={\left(\frac{\sin\;\xi }{\xi }\right)}^{2}\cdot \exp(\frac{-\omega^{2}}{2\;\ln\;2}\xi^{2}),$$ (8)

where $\xi =z/z_{\max}$ is the relative depth normalized to maximum imaging depth and ω=δλ/Δλ represents the ratio of the spectrometer resolution (δλ) and the wavelength spacing between camera pixels (Δλ). A silver mirror was translated at a series of imaging depths and the measured peak reflectance was fit with the above function [Eq. (8)] to confirm the system’s sensitivity roll-off.

### 2.3 Model Coefficient Fitting

A nonlinear least-squares solver was used for estimating the $\mu_{s}$ and $\mu_{b}^{\prime }$ parameters in the nonlinear theoretical model [Eqs. (17)]. The “trusted region reflective” algorithm was used for the optimization process. The trusted region reflective algorithm is an algorithm used in solving large-scale nonlinear equations. Specifically, this algorithm is a subspace adaptation of the Coleman-Li trust region and interior method.^28^ Trust region methods form a respected class of algorithms for solving unconstrained minimization problems mostly due to their strong convergence properties. The algorithm first defines a trust region around the current best solution, then forces the size of the region to be updated iteratively only based on a sufficient decrease in the cost function. All the curve fitting was performed in MATLAB^®^. Before estimating $\mu_{s}$ and $\mu_{b}^{\prime }$ for each position in an image, we first estimate the values of the constant $z_{r}$ and the $z_{f}$ tilt parameterized by Eqs. (2) and (3) by performing a simultaneous fit of OCT depth profiles from multiple positions within a region of the sample where the optical properties are known to be constant. This condition is easily met in phantoms with spatially uniform optical properties. For brain samples where the optical properties are spatially varying, we utilize the agarose material in which our brain tissue is embedded for creating the condition of spatially uniform optical properties for estimating $z_{r}$ and the parameterization of $z_{f}$. The value of $z_{r}$ is then taken to be constant through the entire brain sample, while the value of $z_{f}$ will vary linearly with x and y as prescribed by Eq. (6). Specifically, we fit the depth profiles from multiple locations within the agarose material around the brain tissue simultaneously to extract the value of A, B, and C in Eq. (6). The linear coefficients (B and C) are always constant for different spatial locations due to flattened surface, whereas the constant term (A) is spatially varying due to the angle of the vibratome blade varying from exactly horizontal. Since it is a linear variation, we interpolate the constant term along the direction of surface flattening to obtain the value of A for each image tile.

For comparison with the full model, we fit the four parameters ($\mu_{s}$, $\mu_{b}^{\prime }$, $z_{f}$, and $z_{r}$) independently using Eqs. (2) and (3) for each position within a region. Due to the nonlinearity of the model and the high inter-parameter dependency, a two-step procedure was used as previously described in Ref. 2. We fit the four-parameter model to the averaged experimental data and obtained an average $z_{r}$ for each brain sample. We repeat the fitting with $z_{r}$ fixed to this estimated value and then estimate the spatially varying optical properties and $z_{f}$.

### 2.4 Phantom Experiment Set Up

A set of eight liquid optical phantoms was prepared by diluting different volume concentrations (4%, 8%, 12%, 16%, 20%, 40%, 60%, and 80%) of Intralipid 20% (batch 10LI4282, Fresenius Kabi, Germany) in deionized water to create phantoms with eight different scattering coefficients. Taking into account the properties of the different Intralipid components, pure Intralipid contains 22.7% (v/v) scattering particles (soybean oil and egg lecithin),^29^^–^^32^ the concentration-dependent scattering coefficient can be calculated based on the Twersky quotation and corrected by packing factor^33^

$$\mu_{s}(\lambda ,\phi_{p})=\frac{\mu_{s}^{indep}(\lambda )}{0.227}\phi_{p}\{\frac{{\left(1-\phi_{p}\right)}^{p(\lambda )+1}}{{\left[1+\phi_{p}(p(\lambda )-1)\right]}^{p(\lambda )-1}}\},$$ (9)

where λ is the wavelength, $\phi_{p}$ is the volume concentration of scattering particles in the sample, p is the scattering phase function, and $\mu_{s}^{indep}$ is the independent scattering coefficient. An accurate equation for the independent scattering coefficient of pure intralipid in the 500- to 2250-nm range, was derived in Ref. 34, with $\mu_{s}$ in ${\mathrm{mm}}^{-1}$ and λ in nm.

$$\mu_{s}^{indep}(\lambda )=1.868\times 10^{9}\times \lambda^{-2.59}.$$ (10)

The eight liquid phantoms were imaged with a 10× air objective. Each phantom was measured at 13 different focus depths, starting with $z_{f}=0$ and incrementing by 50  μm up to a depth of 600  μm in the phantom. The three shallow depths ($z_{f}=0$, 50, 100  μm) were discarded from further analysis due to the specular reflection from the phantom surface. The deep depths ($z_{f}>300\;\mu m$) were also discarded due to the poor signal-to-noise ratio causing a constant noise offset in the signal, systematically deviating the experimental data from the theoretical model. Each measurement produces an image that spans over a $1\times 1\times 2.5\;{\mathrm{mm}}^{3}$ volume with a 2.5-μm isotropic pixel size, but with a lateral resolution of 4  μm and an axial resolution of 3.5  μm. During acquisition, A-lines were repeated 10 times to improve the signal to noise ratio. The total acquisition time for each phantom was 13.4 s.

The raw data were resampled in k-space, compensated for dispersion, and Fourier transformed into the spatial domain following standard procedures.^35^ Then the volume data were corrected for sensitivity roll off as described in Ref. 2. Finally, the noise floor, defined as the average intensity at an imaging depth between 2.4 and 2.6 mm where no signal is expected, was subtracted.

### 2.5 Tissue Preparation and Imaging

A human brain tissue was obtained from the Massachusetts General Hospital Autopsy Suite. The brain was from a neurologically normal subject without previous diagnosis of neurological deficits. The sample was fixed with 10% formalin for at least two months. The postmortem interval did not exceed 24 h. The brain was cut into smaller blocks. Then the blocks of interest were embedded in oxidized agarose and covalently cross-linked with the agarose using borohydride borate solution. Embedded human brain blocks were washed for one month in phosphate buffer saline solution (PBS) 0.01 M at room temperature (RT) while gently shaking. Index matching was performed with serial incubations in 100 ml of 20%, 40%, and 60% (v/v) TDE in 0.01 M PBS (TDE/PBS) each for 24 h at RT while gently shaking.

One human brain block from the primary motor cortex was imaged with a 10× air objective (Mitutoyo) and a z-spacer. The surface of the embedded sample was flattened using a vibratome slicer before OCT imaging.^36^ The volumetric imaging spanned a FOV of $1\times 1\times 2.5\;{\mathrm{mm}}^{3}$, with a voxel size of 2.5-μm isotropic. The focus of the light beam was located at roughly 150  μm below the surface. Motorized xyz stages were integrated under the sample arm to automatically translate the sample between image tiles. The $12\times 12\;{\mathrm{mm}}^{2}$ section of the tissue block was imaged with a total of 576 tiles (24×24) that were acquired with 50% overlap between adjacent tiles to ensure reliable registration and to uniformly reduce speckle noise. Fiji software was used to stitch the image tiles and create the mosaic images.^37^

To further reduce the effect of noise on fitting depth-profiles and estimating the optical properties, the volumetric images were averaged over 25  μm in x and y (i.e., 10×10  pixels) and down-sampled in x and y to a 2.5-μm voxel size. The location of the tissue surface was determined on cross-section images using an image processing edge detector.^38^ A total depth range of 250  μm was selected for depth-profile fitting, starting from 35  μm below the tissue surface to reduce the impact of specular reflection from the surface. To compare with conventional OCT contrast, the AIP and maximum intensity projection maps within the same depth range were computed as well.

To further validate our parametrized model, the quantified scattering coefficient of the human brain sample was compared with a conventional low-NA measurement with a negligible axial PSF effect where the OCT signal is modeled as

$$R(z)\propto \exp(-2\mu_{s}z).$$ (11)

We imaged the same section of the brain sample with a 2× air objective (Thorlabs) and a z spacer, which yields a lateral resolution of 15  μm and a theoretical Rayleigh range of 280  μm in tissue. The volumetric image tiles have a FOV of $2\times 2\;{\mathrm{mm}}^{2}$ with a $5\times 5\times 2.5\;\mu m^{3}$ voxel size and 50% overlap between adjacent tiles. A linear function was fit to the logarithmic OCT signal depth profile and the slope was used to extract the $\mu_{s}$ value.

## 3 Results

### 3.1 Optical Properties Characterization of Liquid Phantoms

**Fig. 1:**
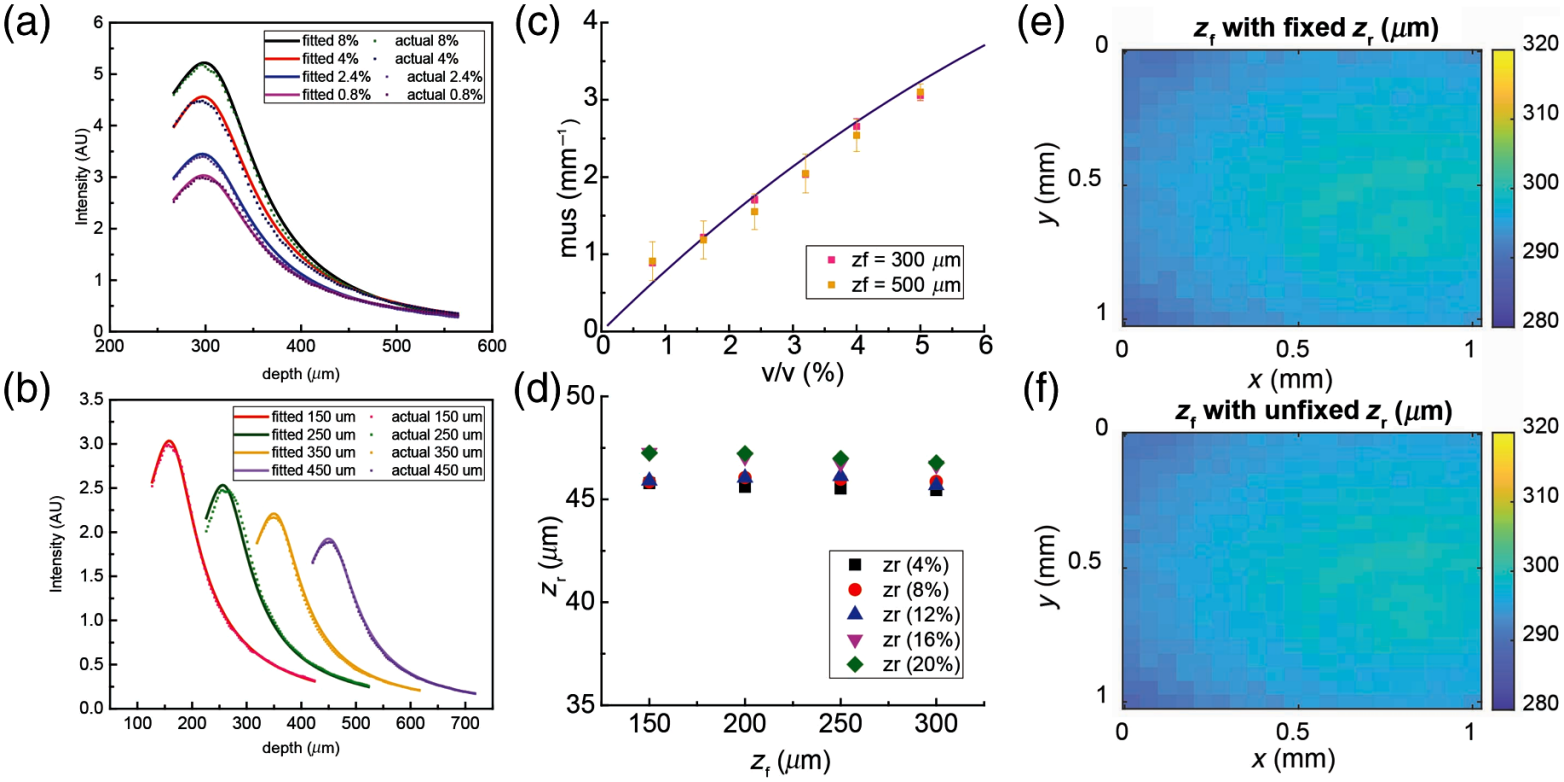
Optical properties of Intralipid liquid phantoms. (a) Representative OCT signal depth profiles and model fitted curves of Intralipid phantoms with different volume concentrations (0.8%, 2.4%, 4%, and 8% v/v) at focus depth of 300  μm. (b) Representative OCT signal depth profiles and model fitted curves of 0.8% v/v Intralipid phantom at different focus depths (150, 250, 350, and 450  μm). (c) Estimation of scattering coefficient ($\mu_{s}$) of Intralipid phantoms with respect to Intralipid volume concentration at two different focus depths. Error bars: standard deviation. The solid line indicates a previous theoretical prediction for the scattering coefficient. (d) Effective Rayleigh range ($z_{r}$) as a function of depth of focus ($z_{f}$) with respect to Intralipid volume concentration. Deep depths of focus were discarded due to the non-negligible effect of multiple scattering. (e) Shape of $z_{f}$ of 0.8% v/v Intralipid phantom within a FOV with fixed $z_{r}$ in model fitting. (f) Shape of $z_{f}$ of 0.8 % v/v Intralipid phantom within a FOV with freely estimated $z_{r}$ in model fitting.

We first fit our parametrized model to the OCT signals acquired from the eight Intralipid phantoms measured at different focus depths. Example fits of the OCT signal depth dependence are shown in Figs. 1(a) and 1(b). In Fig. 1(a), we show example fits of the OCT signal depth profile of liquid phantoms with different volume concentrations (0.8%, 2.4%, 4%, and 8% v/v Intralipid). In Fig. 1(b), we show example fits of the OCT signal depth profile of 0.8% v/v Intralipid phantoms with different focus depths ($z_{f}=150$, 250, 350, and 450  μm). In Fig. 1(c), the estimated scattering coefficient ($\mu_{s}$) values are presented as a function of the volume concentration of Intralipid measured at two different focus depths ($z_{f}$). Error bars indicate the standard deviation of the estimated $\mu_{s}$ across the entire FOV. The error bars are rather small, indicating the high repeatability of the measurements and the coefficient estimation procedure. At high concentration, $\mu_{s}$ tends to be underestimated compared with a prior model of the concentration dependence of $\mu_{s}$ for Intralipid,^32^ probably due to the impact of multiply scattered photons becoming important for higher scattering coefficients.

In Fig. 1(d), the estimated Rayleigh range $z_{r}$ is plotted as a function of both the volume concentration of Intralipid and the focus depth. We found that the estimated $z_{r}$ differed by <1  μm across different focus depths for each volume concentration. This indicated that we could assume $z_{r}$ to be constant across the FOV. We next evaluated the spatial dependence of $z_{f}$ within the FOV both with $z_{r}$ set as a constant and as a freely fitted parameter that was allowed to vary over the FOV, as shown in Figs. 1(e) and 1(f), respectively. We found that the spatial dependence of $z_{f}$ was almost identical with $z_{r}$ set as a constant or as a freely fitted parameter. Moreover, when $z_{r}$ was set as a freely fitted parameter, the coefficient of correlation for the estimated $z_{f}$ and $z_{r}$ was calculated to be −0.043, indicating a strong independence of these two parameters.

**Fig. 2:**
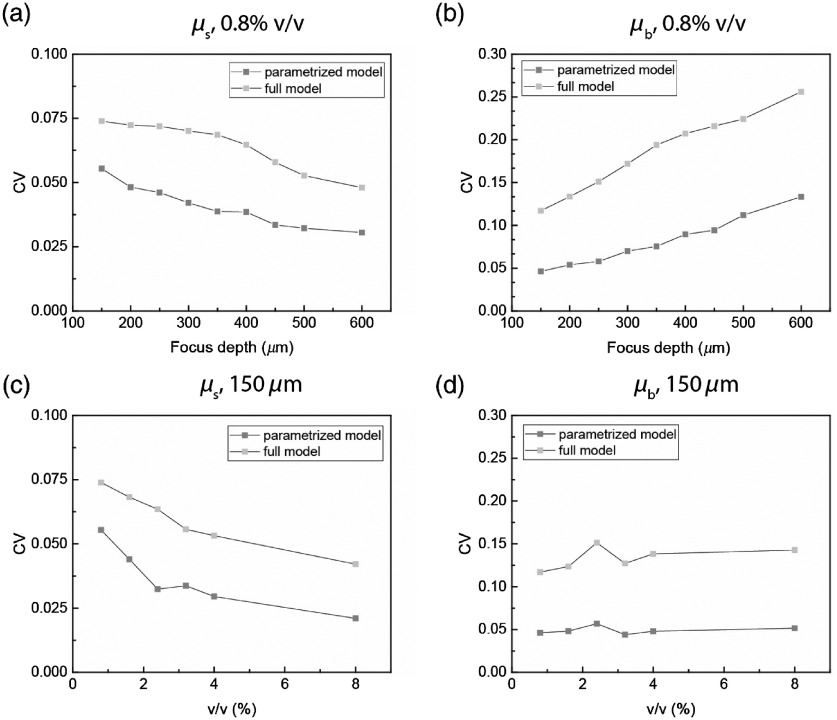
Coefficient of variance (CV) of estimated parameters of liquid phantoms shows improvement of fitting quality. (a) CV of estimated $\mu_{s}$ of 0.8% volume concentration Intralipid phantom over imaging field with various focus depths of both parametrized model and full model. (b) CV of estimated $\mu_{b}$ of 0.8% volume concentration Intralipid phantom over imaging field with various focus depths of both parametrized model and full model. (c) CV of estimated $\mu_{s}$ of Intralipid phantoms at 150-μm focus depth over imaging field with various scattering levels of both parametrized model and full model. (d) CV of estimated $\mu_{b}$ of Intralipid phantoms at 150-μm focus depth over imaging field with various scattering levels of both parametrized model and full model.

To quantify the improvement in fitting quality using the parametrized model compared with the full fitting model, we calculated the coefficient of variance (CV), which is defined as the standard deviation divided by the mean of extracted $\mu_{s}$ and $\mu_{b}^{\prime }$ over the imaging field, as shown in Fig. 2. We first estimated both $\mu_{s}$ and $\mu_{b}^{\prime }$ in an 0.8% volume concentration Intralipid phantom with varying focus depths ($z_{f}$), as shown in Figs. 2(a) and 2(b). We found that our parametrized model showed a smaller CV in both $\mu_{s}$ and $\mu_{b}^{\prime }$ for all focus depths. We then estimated both $\mu_{s}$ and $\mu_{b}^{\prime }$ in a set of liquid phantoms with different concentrations (0.8%, 1.6%, 2.4%, 3.2%, 4%, and 8%) with a fixed focus depth of 150  μm below the liquid surface, as shown in Figs. 2(c) and 2(d). We found that our parametrized model showed a smaller CV in both $\mu_{s}$ and $\mu_{b}^{\prime }$ for all scattering levels.

### 3.2 Optical Properties Characterization of Human Brain

#### 3.2.1 Validation with low-NA model

**Fig. 3:**
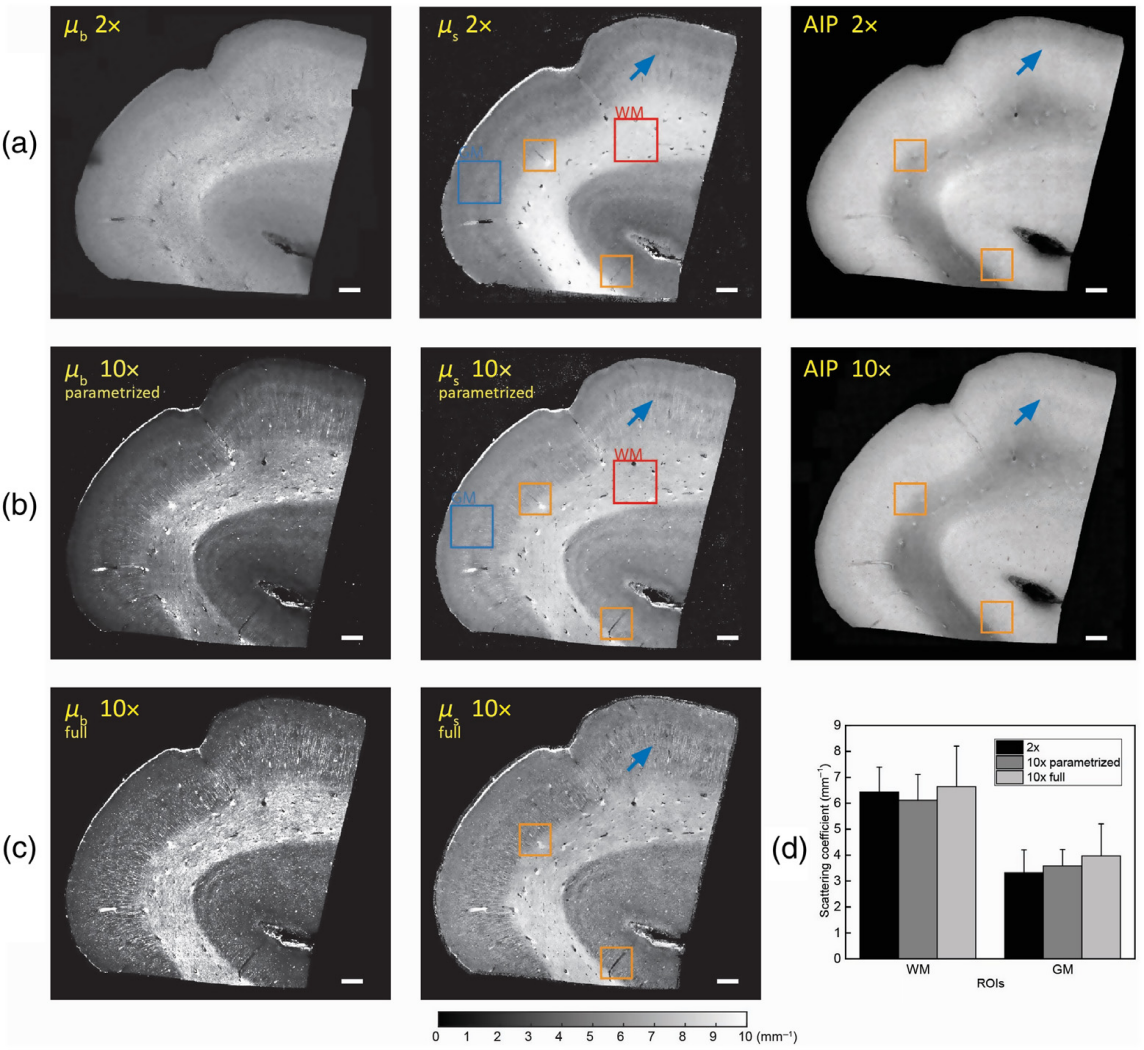
Comparing the scattering coefficient map of primary motor cortex imaged by a low-NA 2× and higher-NA 10× objective. (a) The relative back-scattering (left), scattering coefficient map (middle), and AIP (right) of the brain section with a 2× objective. Some but not all vessels and fiber tracts are labeled with blue arrows. Scale bar: 1 mm. (b) The relative back-scattering (left), scattering coefficient map (middle), and AIP (right) of the brain section with a 10× objective using the parametrized model. Some but not all vessels and fiber tracts are labeled with blue arrows. Scale bar: 1 mm. (c) The relative back-scattering (left) and scattering map (middle) of the brain section with a 10× objective using the full model. Some but not all vessels and fiber tracts are labeled with blue arrows. Scale bar: 1 mm. (d) Averaged $\mu_{s}$ within selected ROIs for both high NA and low-NA objectives. Both parametrized model and full model were used to extract $\mu_{s}$ under high-NA objective.

To validate the quantitative estimates of the optical properties of the brain tissue derived from the proposed parametrized model with the conventional method using a single exponential model, the same section of a human brain sample was imaged with both a high NA (10×, Mitutoyo) and a low-NA (2×, Thorlabs) objective. The impact of axial PSF was negligible for the low-NA objective since the Rayleigh range was longer than the depth range analyzed. We first plot the back-scattering ($\mu_{b}^{\prime }$) and scattering ($\mu_{s}$) maps along with AIPs for both the low-NA and high-NA objectives, as shown in Fig. 3. Moreover, for the high-NA objective, we plot the $\mu_{b}^{\prime }$ and $\mu_{s}$ maps using both the parametrized model [Fig. 3(b)] and the full model [Fig. 3(c)]. To demonstrate the ability of our parametrized model to quantitatively extract the optical properties of human brain tissue, two regions of interest (ROIs) were manually selected, including a $2\times 2\;{\mathrm{mm}}^{2}$ GM region and a $2\times 2\;{\mathrm{mm}}^{2}$ WM region, as shown in Figs. 3(a) and 3(b). The averaged scattering coefficient ($\mu_{s}$) within these two ROIs are plotted in Fig. 3(d). The estimated $\mu_{s}$ of WM were $6.43\pm 0.96\;{\mathrm{mm}}^{-1}$ and $6.12\pm 0.99\;{\mathrm{mm}}^{-1}$ for the low-NA and high-NA objectives, respectively. The estimated $\mu_{s}$ of GM were $3.32\pm 0.89\;{\mathrm{mm}}^{-1}$ and $3.58\pm 0.64\;{\mathrm{mm}}^{-1}$ for the low-NA and high-NA objectives, respectively. We then performed a pairwise t-test on the extracted $\mu_{s}$ from both ROIs using the full and the parametrized models to determine if there is a significant difference in the mean. We found no significant difference in the mean of the estimated $\mu_{s}$(p=0.49 for the GM ROI and p=0.25 for the WM ROI). We further performed a pairwise t-test on the extracted $\mu_{s}$ from both ROIs for the low-NA and high-NA objectives to determine if there is a significant difference in the mean. We found no significant difference in the mean of the estimated $\mu_{s}$(p=0.26 for the GM ROI and p=0.09 for the WM ROI). Although the scattering coefficient is comparable for the two objectives, as expected, we see that structures such as vessels and axonal fiber tracts are more detailed in the high-NA images. As shown in Figs. 3(b) and 3(c), the full four-parameter model overestimated the scattering coefficient of GM and had higher standard deviation within both ROIs compared to the parametrized and the low-NA exponential model. Recall that we take the low-NA measurement as the ground truth result since the model fitting is a simple exponential with no dependence on the confocal parameters.

#### 3.2.2 Comparison with the full fitting model

**Fig. 4:**
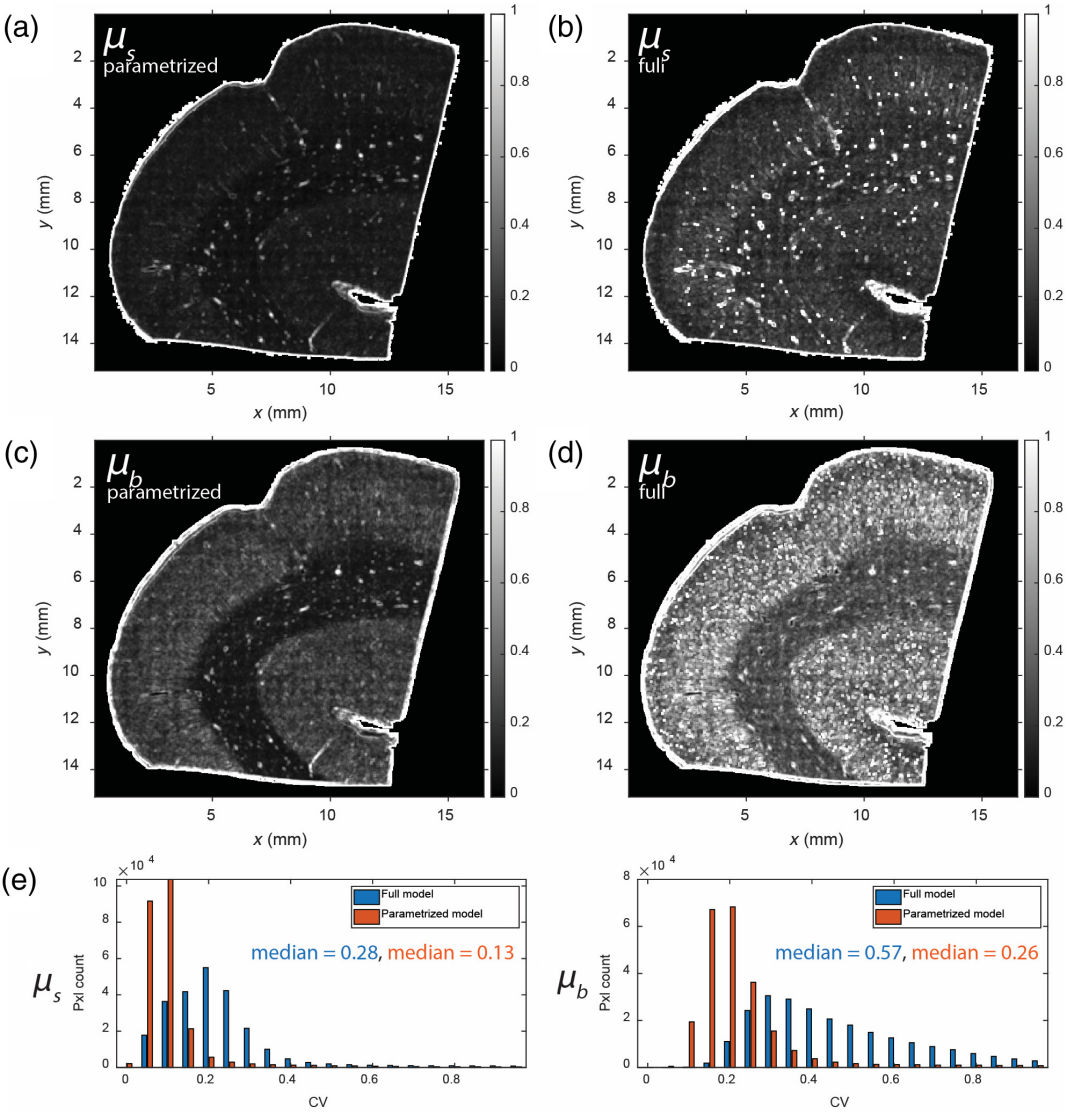
Comparison between the full model and the proposed parametrized model. (a) CV image of the extracted $\mu_{s}$ using the parametrized model. (b) CV image of the extracted $\mu_{s}$ using the full model. (c) CV image of the extracted $\mu_{b}$ using the parametrized model. (d) CV image of the extracted $\mu_{b}$ using the full model. (e) Histograms of CV for the estimated optical properties. Left: CV of the extracted $\mu_{s}$ using both the parametrized and full models. Right: CV of the extracted $\mu_{b}$ using both the parametrized and full models.

We compare the results from our parametrized model with results from the previously established full four-parameter estimation model by imaging the same section of a human motor cortex sample. The same depth range data was used to fit both the full four-parameter model and our proposed parametrized model. To show the improvement for our parametrized model, we generated the CV images for both the back-scattering ($\mu_{b}^{\prime }$) and scattering ($\mu_{s}$) maps for both the parametrized model and the full model using a 5×5  pixel sliding window, as shown in Figs. 4(a) and 4(b). The value of each pixel in the CV images is defined as the standard deviation of values over the sliding window divided by the mean of values over the sliding window. We observed from the CV images that our proposed parametrized model showed a significantly smaller CV compared to the full model. The histograms of the CV values are shown in Fig. 4(d). The median of the CV for $\mu_{s}$ was 0.28 for the full model and was reduced to 0.13 for the parametrized model. Similarly, the median CV of $\mu_{b}^{\prime }$ was 0.57 for the full model and was reduced to 0.26 for the parametrized model. This confirms that our proposed parametrized model is effective at reducing inter-parameter covariation and can provide more precise estimates of the optical properties while maintaining accuracy.

#### 3.2.3 Optical properties of human brain before and after index matching

**Fig. 5:**
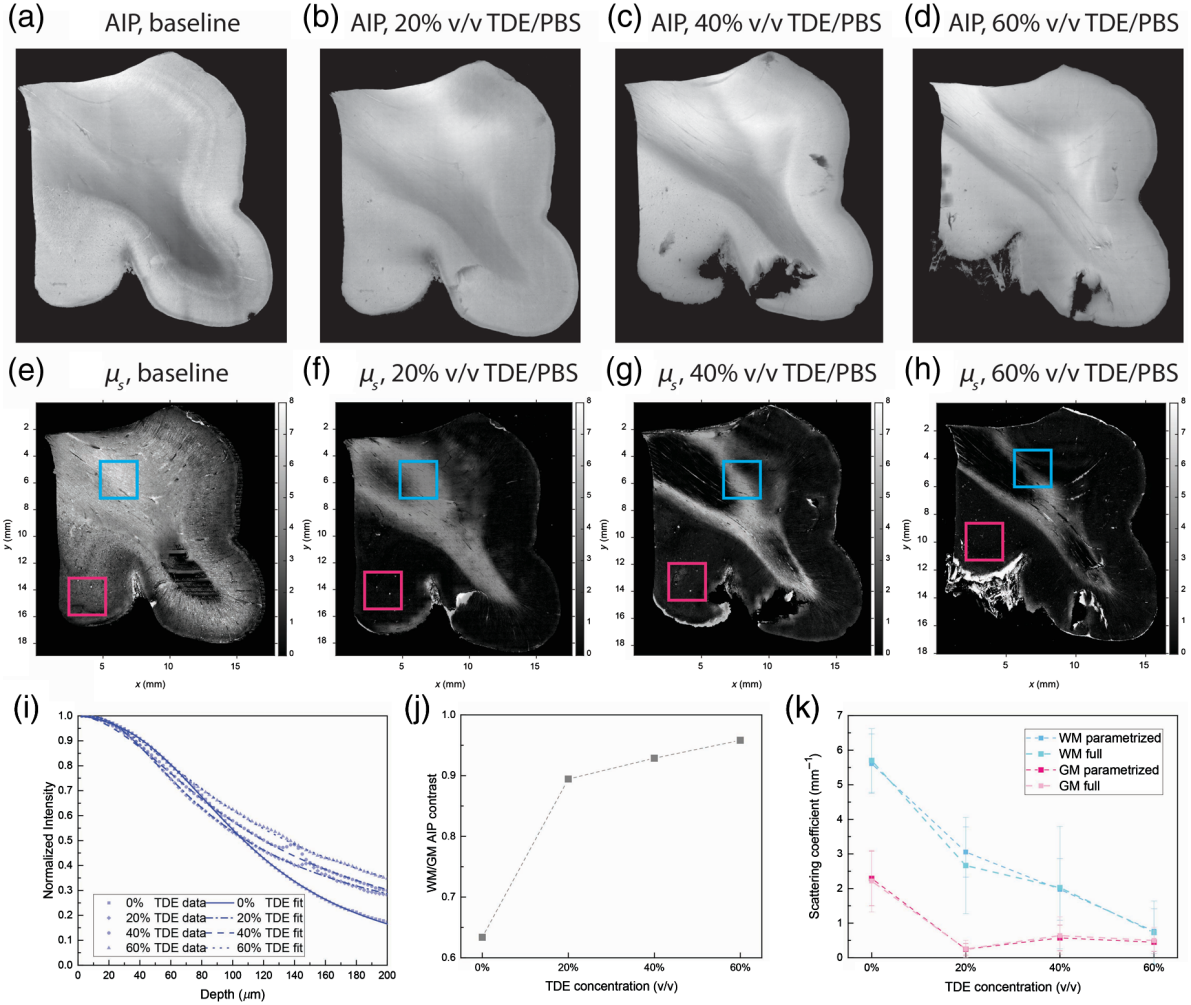
Estimating optical property change during index matching. (a) AIP of the brain section before index matching. (b) AIP of the brain section after equilibrium in 20% v/v TDE/PBS. (c) AIP of the brain section after equilibrium in 40% v/v TDE/PBS. (d) AIP of the brain section after equilibrium in 60% v/v TDE/PBS. (e) Scattering map of the brain section before index matching. (f) Scattering map of the brain section after equilibrium in 20% v/v TDE/PBS. (g) Scattering map of the brain section after equilibrium in 40% v/v TDE/PBS. (h) Scattering map of the brain section after equilibrium in 60% v/v TDE/PBS. (i) Contrast between WM and GM during each TDE concentration. (j) Extracted scattering coefficient from WM and GM ROI during each TDE concentration. Error bar: standard deviation.

A section of a superior frontal cortex brain sample was first surface flattened using a vibratome and imaged with the high-NA objective (10×, Mitutoyo). Then the sample underwent incremental index matching with 20%, 40%, and 60% v/v TDE/PBS. The tissue section was imaged with the high-NA objective at each TDE concentration after it reached equilibrium. Due to nonuniform distortion introduced by index matching, the surface of the tissue block was reflattened at each TDE volume concentration before imaging with the high-NA objective. As shown in Figs. 5(a)–5(d) and 5(j), the AIP indicates a reduced GM/WM contrast after index matching. The GM/WM contrast is defined as the mean value of the WM intensity divided by the mean value of the GM intensity. Prior to index matching, the averaged intensity of WM over the depth range is smaller compared to GM due to the higher scattering coefficient of WM. After index matching, we see that the WM AIP becomes brighter as the ratio between WM and GM intensity reached 0.95 at 60% v/v TDE/PBS equilibrium, suggesting that the index matching process reduced the scattering coefficient of WM more than GM and resulted in a more homogenous scattering level across the sample, as indicated in the extracted $\mu_{s}$ map, as shown in Figs. 5 (e)–5(h).

Two $3.75\times 3.75\;{\mathrm{mm}}^{2}$ (150×150  pixel) ROIs were selected, one from GM and one from WM, as shown in Figs. 5(e)–5(h). The average $\mu_{s}$ of each ROI was calculated to quantify the scattering coefficient change with index matching at each TDE concentration, as shown in Fig. 5(k). We found that after index matching to 60% v/v TDE/PBS, $\mu_{s}$ of GM decreased from $2.29\pm 0.79\;{\mathrm{mm}}^{-1}$ to $0.45\pm 0.27\;{\mathrm{mm}}^{-1}$ and that $\mu_{s}$ of WM decreased from $5.61\pm 0.86\;{\mathrm{mm}}^{-1}$ to $0.77\pm 0.65\;{\mathrm{mm}}^{-1}$. We found that the scattering coefficient of WM decreased to $3.06\pm 0.73\;{\mathrm{mm}}^{-1}$, $1.97\pm 0.89\;{\mathrm{mm}}^{-1}$, and $0.77\pm 0.65\;{\mathrm{mm}}^{-1}$ at 20%, 40%, and 60% v/v TDE/PBS equilibrium, respectively, whereas the scattering coefficient of GM decreased to $0.25\pm 0.17\;{\mathrm{mm}}^{-1}$ and $0.57\pm 0.37\;{\mathrm{mm}}^{-1}$ at 20% and 40% v/v TDE/PBS equilibrium, respectively, and then reached $0.45\pm 0.27\;{\mathrm{mm}}^{-1}$ at 60% v/v TDE/PBS equilibrium. The different behavior of GM and WM under different TDE concentration index matching is a result of the expected lower average index of refraction for GM relative to WM.^39^ To better justify the use of the parametrized model, we also fitted the scattering coefficient at each TDE concentration with the full model, as shown in Fig. 5(k). We found that, in general, both models agreed with each other on the extracted scattering coefficient whereas the standard deviation of estimated $\mu_{s}$ was smaller for the parametrized model at each TDE concentration.

## 4 Discussion

Previous studies investigating the optical properties of biological tissues with OCT primarily adopted a simple exponential model based on Beer’s law.^40^ For high-resolution OCT, models including the impact of confocal parameters have been proposed, but with high inter-parameter correlation, which resulted in large variance in the extracted coefficients.^2^ Here, we extended this prior model by developing procedures to spatially parametrize the confocal parameters to reduce the degrees of freedom of the fitting model and produce estimated values with less variance compared with the prior full four-parameter model. We have detailed a straight-forward approach of utilizing field curvature correction, estimating the constant Rayleigh range, and parameterizing the focus depth over the FOV to achieve more precision in the estimated optical properties of the sample while maintaining accuracy. We anticipate this parameterized model being used routinely for estimating tissue optical properties with high-NA OCT measurements.

There are a few remaining limitations in this revised fitting model. The refractive index difference at the liquid-tissue interface was not considered in the axial PSF model. Both Schmitt et al.^4^ and Izatt et al.^11^ have derived PSF models that include this effect. However, these models would require more parameters and could introduce inter-parameter dependency, which could potentially cause greater bias in the prediction. However, it was reported previously that the estimated $\mu_{s}$ of microsphere suspensions using the Izatt model and our model had no significant difference,^2^ suggesting that this effect is negligible. Further support for this comes from agreement in our estimated values using low-NA measurements, which are not impacted by the index of refraction mismatch, and the high-NA measurements. An additional potential limitation is that we did not consider differences in the index of refraction for different tissue types, which could cause variations in $z_{f}$ and $z_{r}$ within the FOV. Our current model assumed a single index of refraction in the tissue and constrained its effect on the axial PSF. Neglecting this effect introduces some uncertainty in the estimate of the optical properties. In our OCT reflectance model, the refractive index (n) plays a role in two ways: the effective Rayleigh range ($z_{r}=n\cdot z_{R}$) and the axial step size. Uncertainty in each of these will introduce uncertainty in the estimated scattering coefficient. In our proposed approach, the effective Rayleigh range was estimated directly from the depth profile of the experimental data, and thus would not be affected by uncertainty in the refractive index. But uncertainty in the refractive index will produce uncertainty in the axial step size. To quantify the impact of this uncertainty, we compared the scattering coefficient extracted using the refractive index of WM (n=1.45) and that of water (n=1.33), which are extreme possible values for the refractive index. We selected an ROI of $1\;{\mathrm{mm}}^{2}$ from within the red ROI shown on the human WM sample shown in Fig. 3 and performed coefficient estimation with our parametrized method. We found that the estimated scattering coefficient varied by ∼8%, which is slightly <9% change in the refractive index. It makes sense that the uncertainty in the estimated scattering coefficient scales directly with the systematic error in the index of refraction as this changes the axial length scale on which the scattering coefficient is estimated. Overall, this uncertainty is smaller than the standard deviation we observed in selected ROIs, as shown in Fig. 3(d), which varied from 17.9% to 31.1%. A third limitation is that we assumed negligible contributions from multiple scattering of light within the sample. Given that we are constraining our estimates to relatively shallow depth ranges, we are comfortable with this assumption. We do note that the effect of multiple light scattering likely revealed itself in our phantom measurements at high scattering coefficients.

A future goal for advancing this fitting model is to remove the assumption of constant optical properties over the fitting depth range and expand the model to estimate a depth-dependent scattering coefficient. Achieving this would provide 3-D maps of the tissue optical properties with high-NA OCT and greatly improve the impact for varied applications. Given that depth-resolved models have been developed for low-NA OCT and have been implemented in retinal, colon, and bladder imaging,^41^^,^^42^ we anticipate future efforts will succeed in doing the same with high NA OCT.

With this new spatially parameterized fitting model, we performed a preliminary analysis of the impact of a tissue index matching procedure on the quantitative scattering coefficient of the tissue. Our preliminary result quantified the reduction in scattering in GM and WM caused by increasing the concentration of the index matching agent TDE. It was previously reported that index matching with TDE increased the transmittance in fixed brain slices in a concentration-dependent manner, leading to significant enhancement of the penetration depth.^43^ We found that 60% TDE increased the scattering length in the GM/WM by 370.6%/294.4% compared to no index matching. In the case of no index matching, we note that our scattering coefficient was less than previously reported,^2^ but this can be explained by our washing samples with PBS versus the prior work where the tissue was emersed in fixative, which is known to enhance optical scattering.^44^ Interestingly, we observed that a minimum in the GM scattering coefficient was obtained with 20% to 40% TDE and that GM scattering increased at 60% TDE while WM scattering continued to decrease. This is consistent with reports that the GM index of refraction is less than that of WM.^39^ Specifically, the index of refraction for GM has been reported to be 1.367, which matches a TDE concentration of about 30% while the index of refraction for WM has been reported to be 1.467, which matches a TDE concentration of about 65%.^21^ Future work performing a more in-depth analysis expanding on our preliminary result will help identify the optimal concentration of the index matching agent to use for maximizing image features and contrast of interest.

## Code, Data, and Materials Availability

The code associated with the manuscript is available and will be uploaded to Github repository. The data and materials associated with the manuscript are also available per request.
